# Author Correction: ANKS1A regulates LDL receptor-related protein 1 (LRP1)-mediated cerebrovascular clearance in brain endothelial cells

**DOI:** 10.1038/s41467-024-47249-w

**Published:** 2024-04-09

**Authors:** Jiyeon Lee, Haeryung Lee, Hyein Lee, Miram Shin, Min-Gi Shin, Jinsoo Seo, Eun Jeong Lee, Sun Ah Park, Soochul Park

**Affiliations:** 1 Department of Biological Sciences, Sookmyung Women’s University, Seoul, 04310 Korea; 2 Department of Brain Sciences, Daegu Gyeongbuk Institute of Science & Technology (DGIST), Daegu, 42988 Korea; 3 Department of Brain Science, Ajou University School of Medicine, Suwon, 16499 Korea; 4 Lab for Neurodegenerative Dementia, Department of Anatomy, and Department of Neurology, Ajou University School of Medicine, Suwon, 16499 Korea

**Keywords:** Blood-brain barrier, Alzheimer's disease

Correction to: *Nature Communications* 10.1038/s41467-023-44319-3, published online 20 December 2023

The original version of this Article contained incorrect microscopy images in Fig. 3c. In the original Fig. 3, the bottom row right immunofluorescence image in Fig. 3c (the representative image for hippocampus for ANKS1Af/f:Tie2-Cre mice) was inadvertently duplicated from the bottom row middle panel of 3c (the representative image for hippocampus for ANKS1Af/f mice).

The correct version of Fig. 3 is:
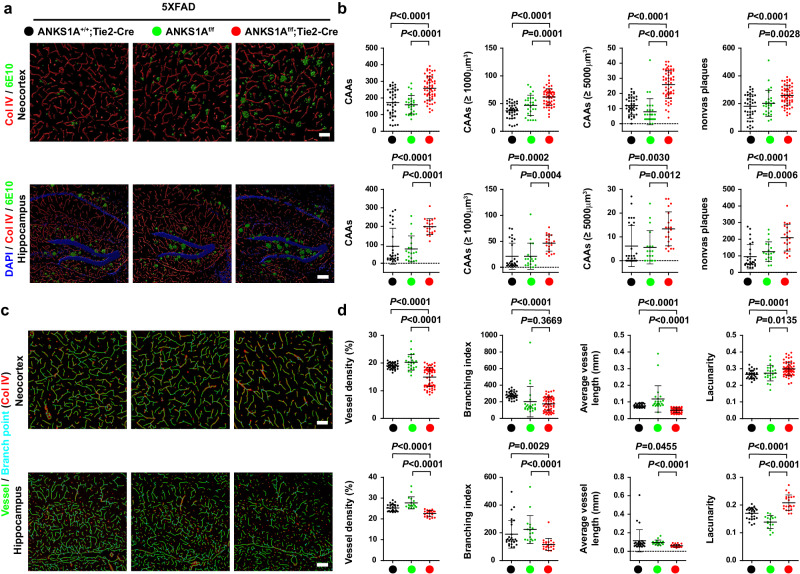


Which replaces the previous incorrect version:
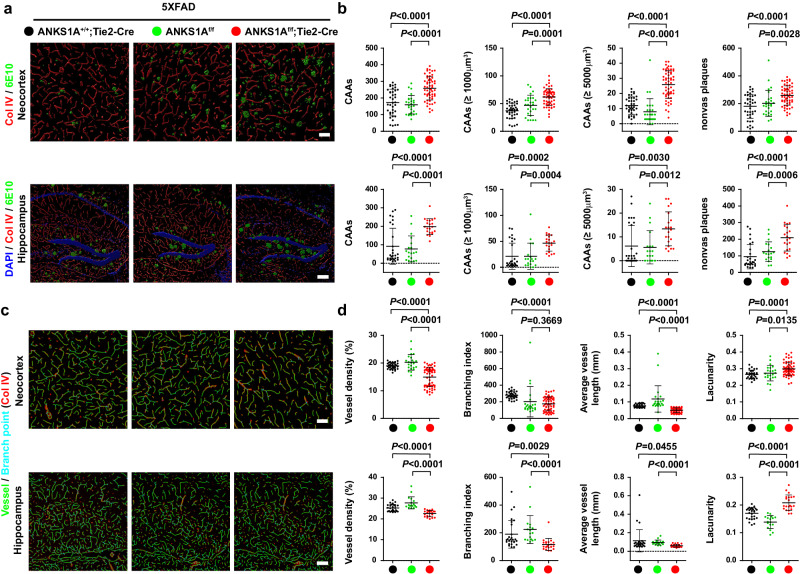


Figure 3 has been corrected in both the PDF and HTML versions of the Article.

