# Supplementary material for: ANKS1A regulates LDL receptor-related protein 1 (LRP1)-mediated cerebrovascular clearance in brain endothelial cells
Source: Nat Commun. 2023 Dec 20;14:8463. doi: 10.1038/s41467-023-44319-3 (PMC10733300; doi:10.1038/s41467-023-44319-3)
Supplement: Supplementary file 1 — Supplementary Information [file 41467_2023_44319_MOESM1_ESM.pdf]

Supplementary Information

ANKS1A regulates LDL receptor-related protein 1 (LRP1)-mediated cerebrovascular clearance in brain endothelial cells

Jiyeon Lee[1, #], Haeryung Lee[1, #], Hyein Lee[2], Miram Shin[1], Min-Gi Shin[3], Jinsoo Seo[2], Eunjeong Lee[3], Sun Ah Park[4], and Soochul Park[1, *]

[1] Department of Biological Sciences, Sookmyung Women's University, Seoul 04310, Korea; [2] Department of Brain Sciences, Daegu Gyeongbuk Institute of Science & Technology (DGIST), Daegu 42988, Korea; [3] Department of Brain Sciences, Ajou University School of Medicine, Suwon 16499, Korea; [4] Lab for Neurodegenerative Dementia, Department of Anatomy, and Department of Neurology, Ajou University School of Medicine, Suwon 16499, Korea

[#] These authors contributed equally to this work.

* Correspondence and request for materials should be addressed to S.P.

(email: scpark@sookmyung.ac.kr).

Running title : ANKS1A regulates cerebrovascular Aβ clearance

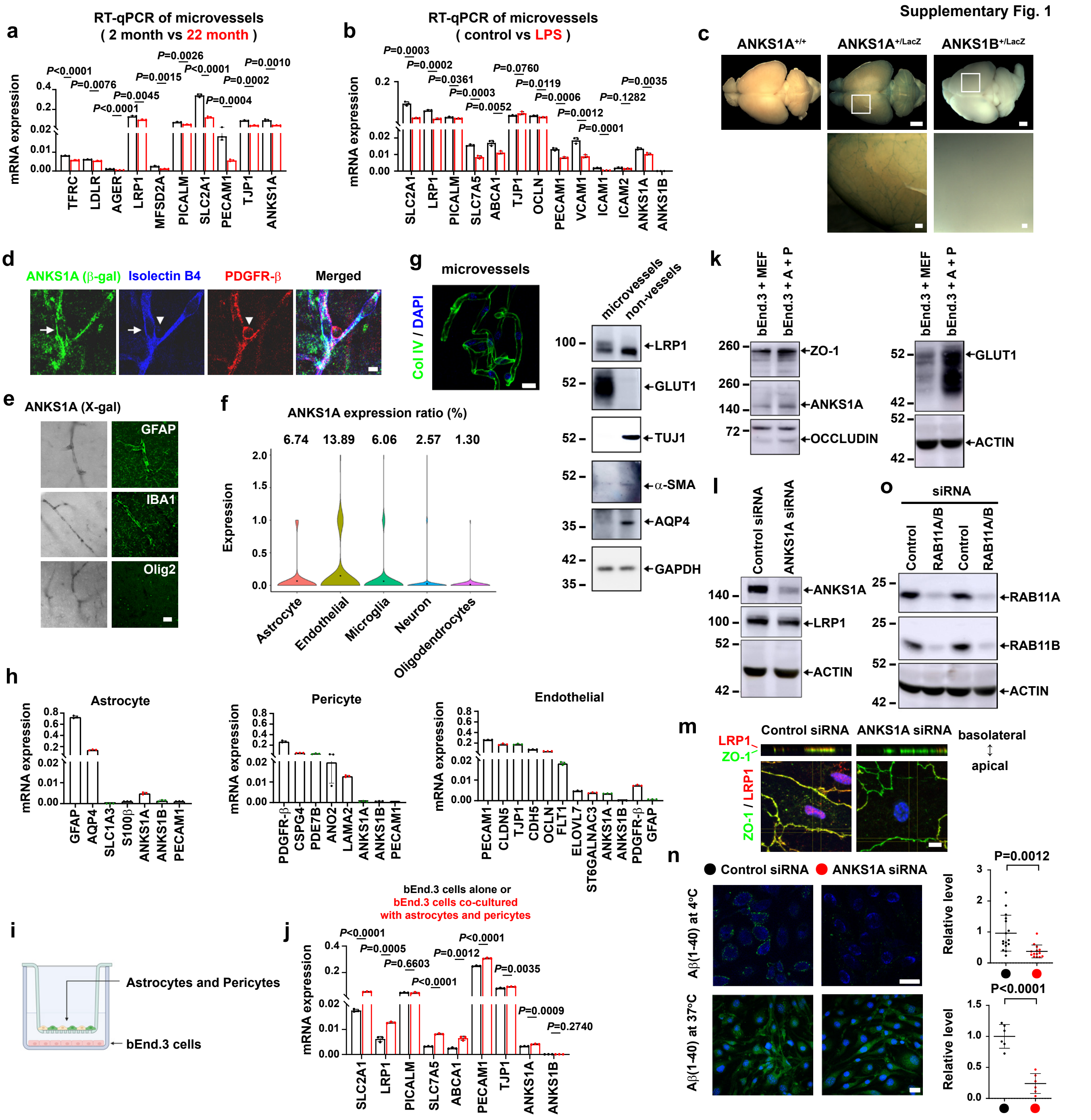

Supplementary Figure 1. Brain endothelial expression of *ANKS1A* is regulated by the integrity of BBB (related to Fig. 1).

(a) Comparative RT-qPCR analysis of samples from the 2 month-old versus the 22 month-old mice. Expressions were normalized to GAPDH. N=3 mice for each group. **(b)** Comparative RT-qPCR analysis of samples from control versus LPS-treated adult mice. N=3 for each group. **(c)** X-gal staining of whole brains from the indicated mice (scale bars, 2 mm). **(d)** Immunohistochemical staining for the X-gal stained microvessels. *ANKS1A*-expressing cell is co-localized with the IB4-positive cell (an arrow) rather than the PDGFRβ-positive cells (an arrowhead) (scale bar, 5 μm). **(e)** The X-gal stained microvessels were co-stained with the indicated markers (scale bar, 20 μm). **(f)** The expression levels of *ANKS1A* in various cell types of the hippocampus were analyzed using the published snRNaseq dataset (GSE 166261). **(g)** Isolated microvessels were analyzed by both immunostaining and western blot analysis (scale bar, 10 μm). **(h)** Each primary cell constituting the BBB was analyzed for the cell-type specific gene expression using RT-qPCR. **(i)** Schematics showing the co-culture of bEnd.3 cells with mouse primary pericytes and astrocytes. **(j)** Comparative RT-qPCR analysis of control bEnd.3 cells versus soluble factor-exposed bEnd.3 cells. **(k-o)** bEnd.3 cells were cultured as in Supplementary Fig. 1i. **(k, l, o)** Western blot analysis was performed on the cell lysates using the indicated antibodies. **(m)** The localization of LRP1 to the basolateral side of bEnd.3 cells was analyzed under unpermeabilized conditions. Top panels show z-stack confocal images of the regions marked with two lines (scale bar, 8 μm). **(n)** For the cell surface binding assay, the HyLite488-labeled Aβ(1-40) peptide was added to cells at 4 °C for 4 hours. For the internalization experiment, the Aβ(1-40) peptide was added to cells at 37 °C for 15 min. *n*=16, 14 for each group (upper panel); *n*=6 for each group (lower panel). Scale bar, 20 μm. Results (c, d, e, g, k, l, m, o) were reproduced at least three independent experiments. Data (h, j, n) were from three independent experiments. Data in this figure are shown as mean ± SD. Two tailed unpaired t-test. The illustration in panel i was created with Biorender. com

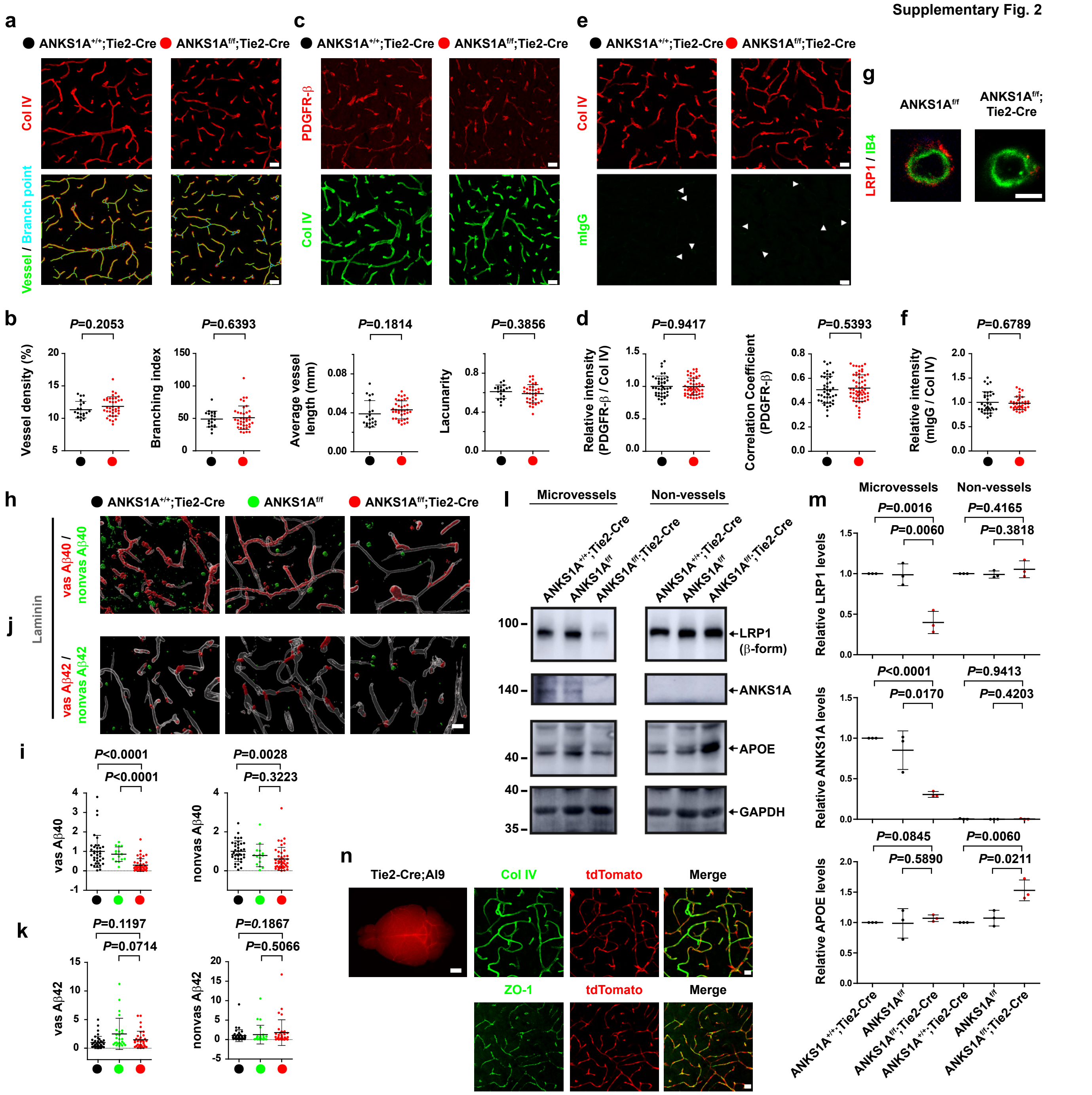

Supplementary Figure 2. Endothelial-specific *ANKS1A* loss does not disrupt the cerebrovascular integrity and BBB functionality in the mouse brain (related to Fig. 2). **(a)** Fixed brains from each mice at 2 months of age were cut into serial coronal sections. The confocal images were rendered into a vascular network image (scale bar, 20 μm). **(b)** Data were quantified for vessel density, branching points, average length and lacunarity (N=3 mice, *n*=20, 27 or 28 sections for each group). **(c)** Immunohistochemical staining was performed using the vessel-specific Col IV, and the pericyte-specific PDGFR-β (scale bar, 20 μm). **(d)** The intensity of PDGFR-β staining was normalized to that of Col IV staining, and the Pearson Correlation Coefficient was calculated (N=3 mice; *n*=41, 52 for each group (left panel); *n*=43, 61 for each group (right panel)). **(e)** Immunohistochemical staining was performed using Col IV, and peripheral blood, mouse IgG, antibody markers (scale bar, 20 μm). **(f)** Mouse IgG staining intensity was normalized to that of Col IV staining. The arrowheads indicate weak IgG signals. N=3 per group, *n*=34, 37 sections for each group. **(g)** Using antibodies directed against IB4 (luminal side) and LRP1 (abluminal side), the polarized expression of LRP1 to the abluminal side was analyzed in brain microvessels (scale bar, 5 μm). **(h-k)** The hippocampal sections were analyzed. (scale bar, 30 μm). **(i)** *Tie2-Cre* group, N=5, *n*=36; *ANKS1A^f/f^* group, N=3, *n*=15; *ANKS1A^f/f^;Tie2-Cre* group, N=5, *n*=44. **(k)** *Tie2-Cre* group, N=5, *n*= 38; *ANKS1A^f/f^* group, N=3, *n*=25; *ANKS1A^f/f^;Tie2-Cre* group, N=5, *n*=34. **(l, m)** Western blot analysis was performed on microvessel or nonvascular extracts. The intensity of each protein was divided by that of GAPDH for normalization. N=3 for each group from three independent experiments. **(n)** The reporter expression of *Tie2-Cre;Ai9* brain was analyzed by immunostaining. Scale bar for whole brain, 1 mm; scale bar for sections, 20 μm. Results (g, n) were reproduced in three independent experiments. Data in this figure are represented as mean ± SD. Two tailed unpaired t-test against *ANKS1A^f/f^;Tie2-Cre* populations.

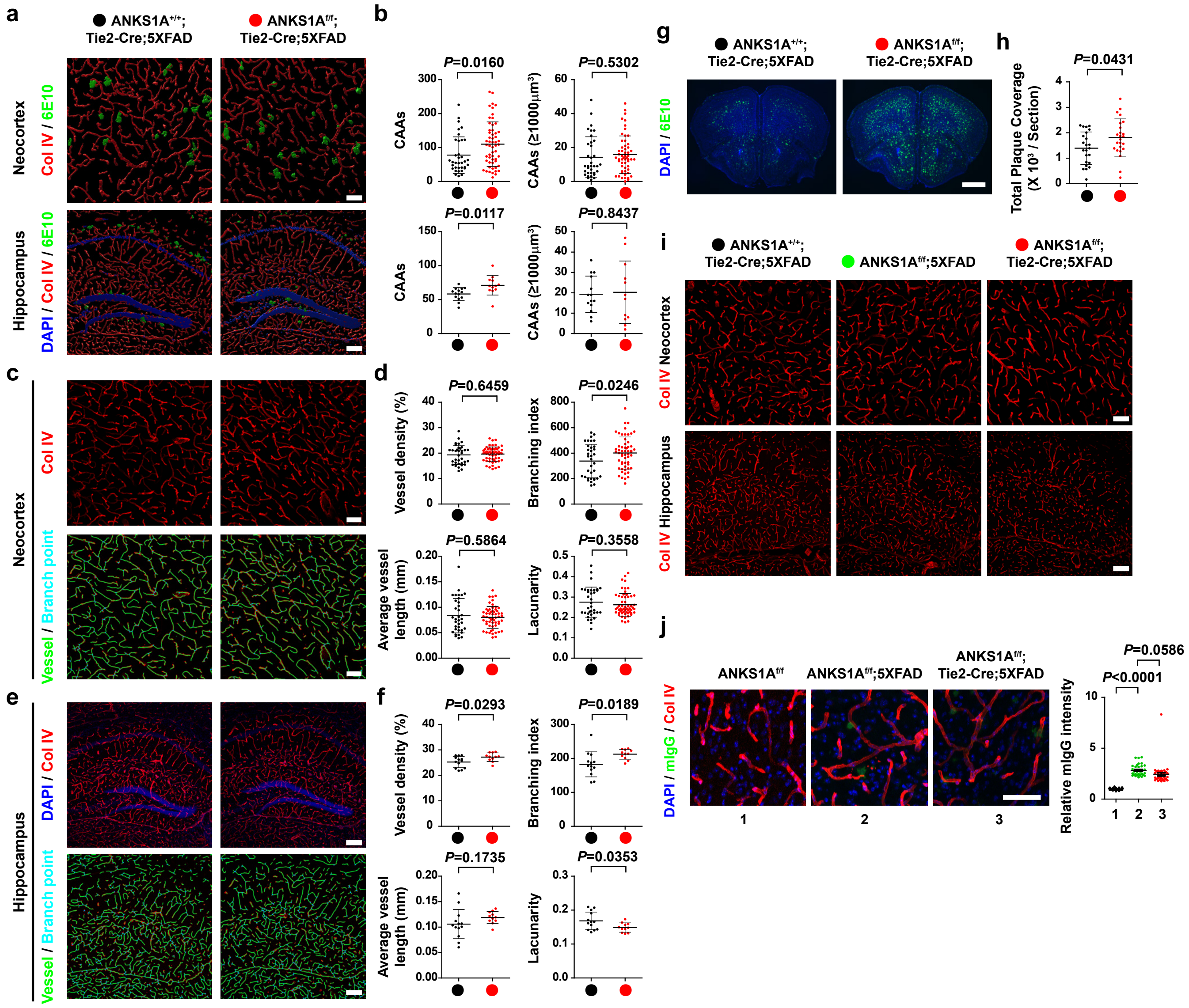

Supplementary Figure 3. Endothelial-specific *ANKS1A* loss exerts a marginal effect on the AD-related pathology in 5XFAD mice at 5 months of age (related to Fig. 3). Experiments were performed as described in Fig. 3, except that the data were obtained from 5 month-old mice. **(a, b)** Immunohistochemical staining using the 6E10 antibody and quantifications were performed (scale bars, 50 μm (neocortex) and 100 μm (hippocampus)). N=3 mice, *n*=35, 56 sections for each group (upper panels); N=3 mice, *n*=14, 12 for each group (lower panels). **(c-f)** Vascular patterning analysis was performed (scale bar, 50 μm (neocortex), 100 μm (hippocampus)). **(d)** N=3 mice, *n*=35, 57 for each group, **(f)** *n*=13, 11 for each group. **(g-j)** The sections were prepared from 7 month-old mice. **(g, h)** The total numbers for 6E10-positive puncta in each section were counted and are presented as total plaque coverage (scale bar, 500 μm). N=3 mice, *n*=13, 14 for each group. **(i)** Confocal images of Col IV-stained sections in Fig. 3c are shown (scale bars, 50 μm (neocortex) and 100 μm (hippocampus)). **(j)** Experiments were performed as described in Supplementary Fig. 2e, f (scale bar, 50 μm). N=3 mice, *n*=28, 36, 38 for each group. Data in this figure are shown as mean ± SD. Two tailed unpaired t-test.

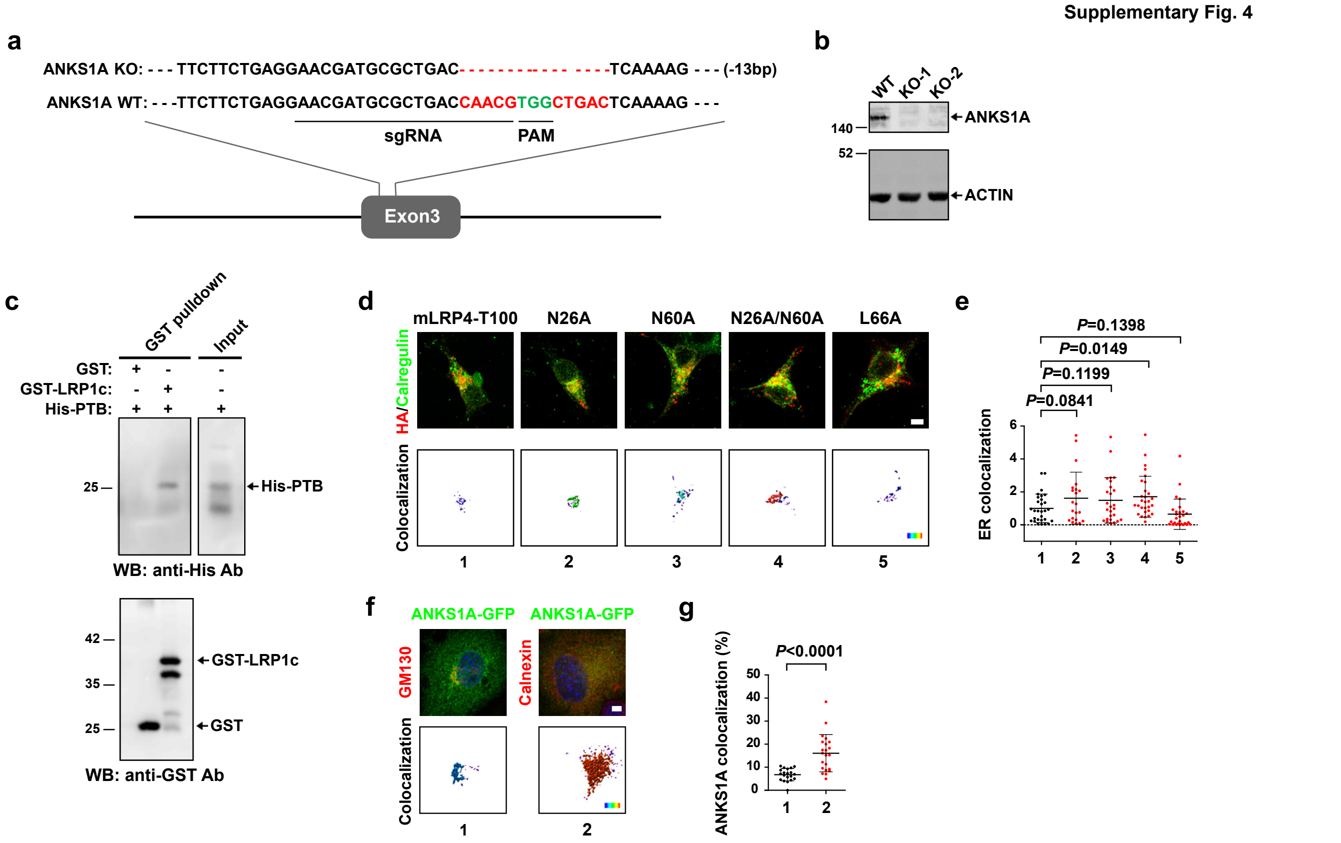

Supplementary Figure 4. ANKS1A plays a role in facilitating the export of LRP1 from the ER in HEK293 cells (related to Fig. 4).
(a) Schematic diagram depicting the CRISPR/Cas9 editing strategy for *ANKS1A* KO in HEK293 cells. **(b)** Western blot analysis showing that endogenous ANKS1A was absent in two independent KO HEK293 cell lines. **(c)** The GST-tagged cytoplasmic LRP1 and His-tagged *ANKS1A*-PTB domain fusion proteins were purified and then GST-pulldown experiments were performed to study the direct interaction between the two sets of proteins. **(d, e)** The experiments were performed as described in Fig. 4h, i (scale bar, 5 μm). *n*=29, 21, 26, 29 cells for each group. **(f, g)** bEnd.3 cells were transfected with the *ANKS1A*-GFP construct and then the ectopic GFP expression was analyzed with the Golgi-marker anti-GM130 and ER-marker anti-calnexin antibodies, respectively (scale bar, 5 μm). The fraction of ANKS1A co-localized in each compartment were measured. *n*=20, 22 for each group. Results (b, c) were confirmed at least in three independent experiments. Data (e, g) were from three independent experiments. Data in this figure are shown as mean ± SD. Two tailed unpaired t-test against mLRP4-T100 populations.

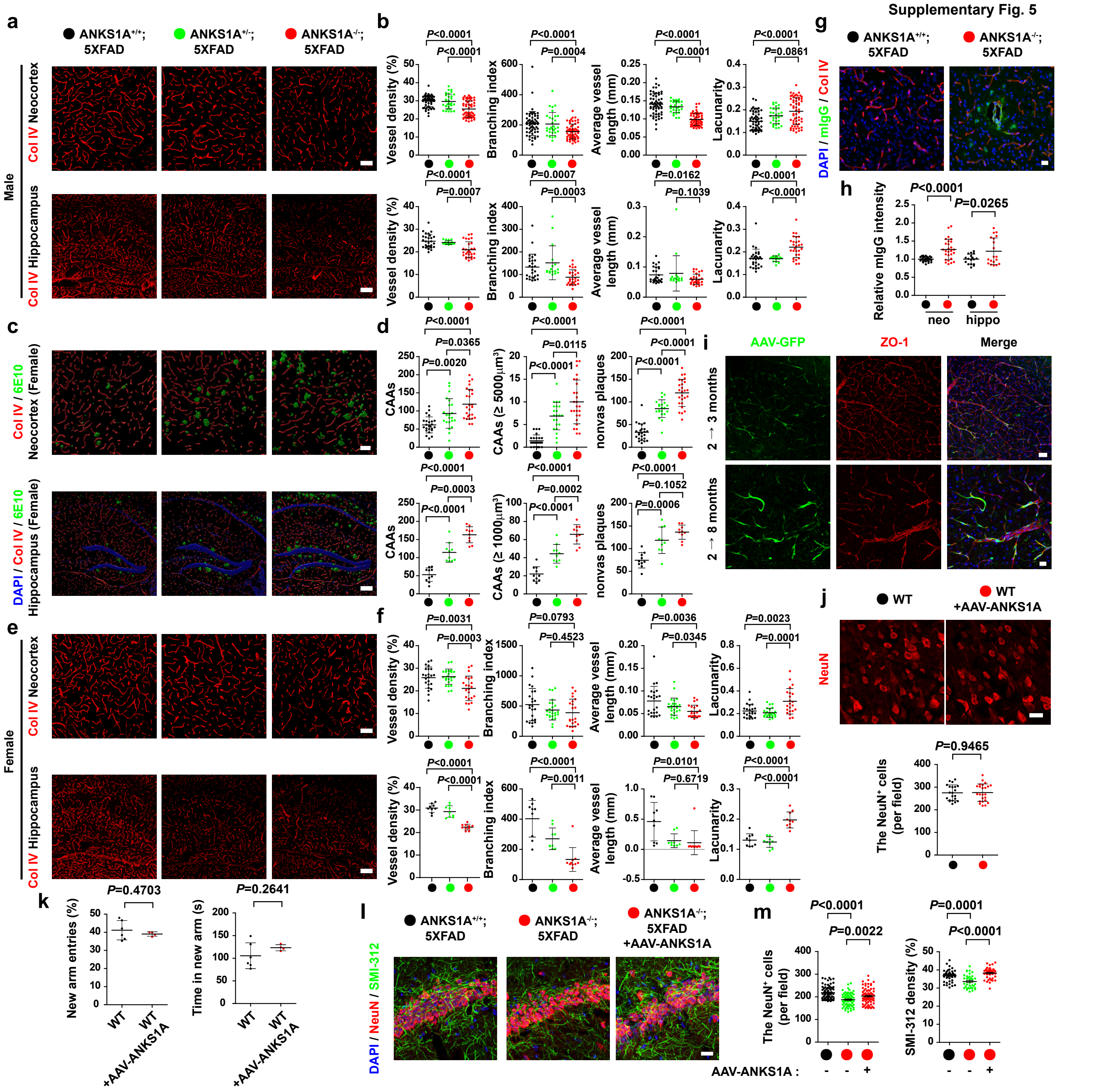

Supplementary Figure 5. Conventional ANKS1A KO exerts a greater effect on the AD-related pathology of 5XFAD mice at 5 months of age (related to Fig. 5).
(a, b) Vascular patterning analysis (scale bars, 50 μm (neocortex) and 100 μm (hippocampus)) (N=5, N=3, N=5 mice for each group; *n*=58, 30, 59 sections for each group (upper panels); n=28, 17, 28 for each group (lower panels)). **(c, d)** Female mice at 5 months of age were used (N=3 for each group; *n*=24, 22, 26 for each group (upper panels); *n*=10 for each group (lower panels). **(e, f)** Vascular patterning analysis using the female mice (N=3 for each group; *n*=23, 25, 22 for each group (upper panels); *n*=9, 9, 10 for each group (lower panels)). **(g, h)** Peripheral blood leakage analysis was performed (scale bar, 20 μm). N=3 mice for each group; neocortex, *n*=27 for each group; hippocampus, *n*=17, 19 for each group. **(i)** *AAV-BR1-GFP* virus was injected into C56BL/6 mice at 2 months of age; the injected mice were sacrificed one month or six months later for GFP expression analysis (scale bar, 50 μm). **(j)** *AAV2-BR1-ANKS1A* was injected into WT mice and then analyzed together with the uninjected WT mice at 5 months of age. The NeuN-positive neurons in the neocortical layer V were quantified (scale bar, 20 μm). Each dot represents the number of NeuN-positive neurons in each field. N=6 for WT, N=4 for injected mice; *n*=21, 24 sections for each group. **(k)** The modified Y-maze test for mice. N=6, 4 mice for each group. **(l, m)** The experiments were performed as described in Fig. 5e, f, except that we analyzed the hippocampus (CA1) (scale bar, 20 μm). N=3 mice for easch group; *n*=72, 72, 73 for each group (left panel); *n*=42, 38, 45 for each group (right panel). Data in this figure are shown as mean ± SD. Two tailed unpaired t-test.

Supplementary Fig. 6

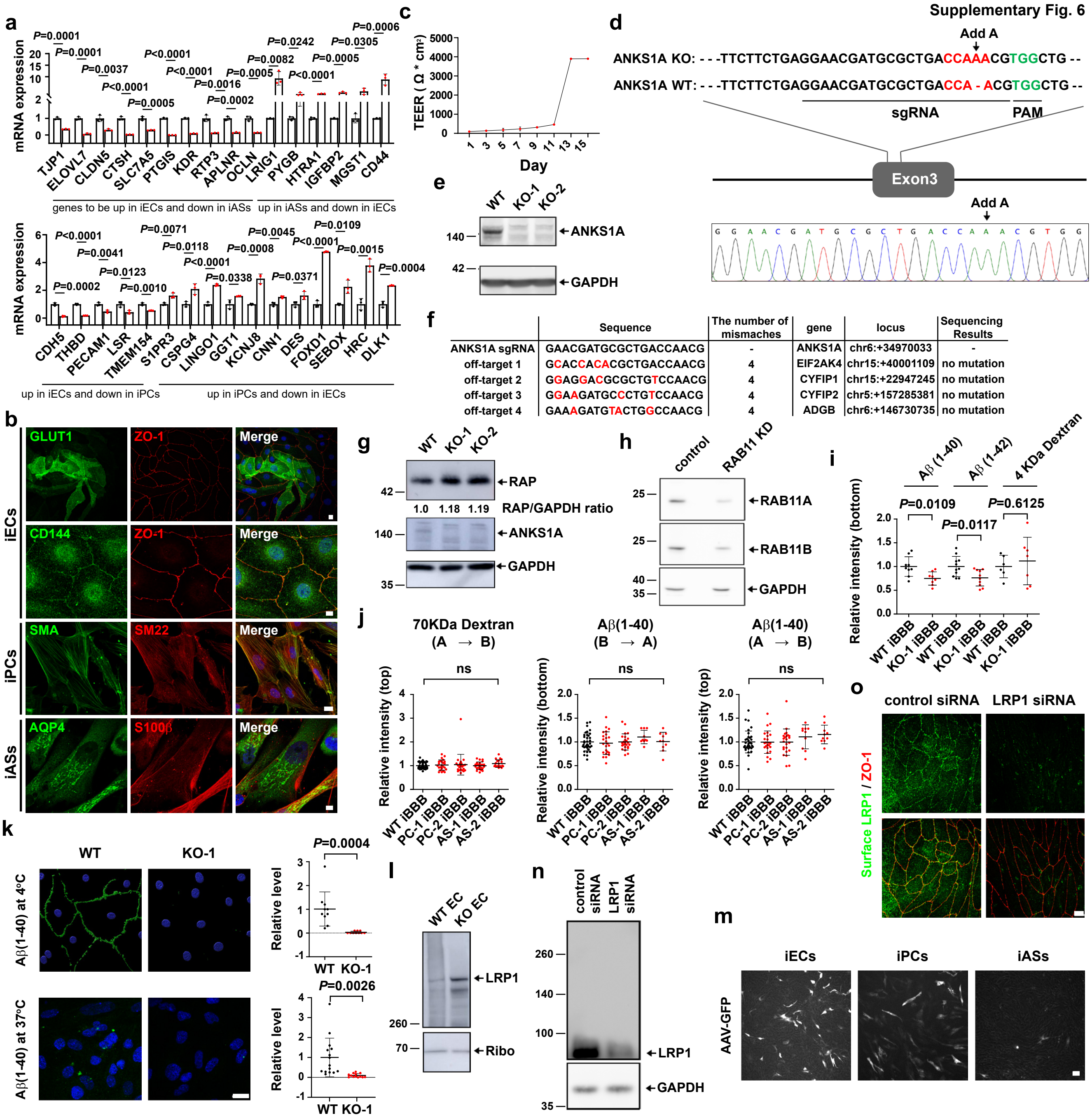

Supplementary Figure 6. Human iPSCs are differentiated into the cell types constituting the BBB (related to Fig. 6).

The iPSC cells used in this figure were derived from the GM23720 cell line. **(a)** Comparative RT-qPCR analysis of the differentially upregulated genes in the respective cells. **(b)** iPSC-differentiated cells were stained with the indicated antibodies (scale bar, 20 μm). **(c)** Changes in TEER values in WT iBBBs over time. **(d, e, f)** Generation of isogenic *ANKS1A* KO iPSCs using the CRISPR/Cas9 gene editing, confirmation of protein expression and validation with the potential off-targets. **(g)** Western blot analysis to determine the levels of RAP. **(h)** Western blot analysis to determine the levels of RAB11A/B in control or *RAB11A/B* siRNA-transfected iECs. **(i)** *n*=9, 8 wells for each group (first panel); *n*=11, 10 for each group (second panel); *n*=6, 7 for each group (third panel). **(j)** Paracellular permeability/transcytosis assay. Note that each iBBB contained iECs expressing WT ANKS1A. *n*=42, 24, 24, 24, 20 wells for each group (first panel); *n*=36, 24, 24, 10, 10 for each group (second panel); *n*=34, 24, 24, 10, 10 for each group (third panel). **(k)** For the cell surface binding assay, the HyLite488-labeled Aβ(1-40) peptide was added to cells at 4 °C for 4 hours. For the internalization experiment, the Aβ(1-40) peptide was added to cells at 37 °C for 15 min (scale bar, 20 μm). *n*=10 for each group (upper panel); *n*=15, 13 for each group (lower panel). **(l)** Digitonin-permeabilization was used to obtain crude ER extracts, followed by western blot analysis. LRP1 levels were normalized with ribophorin levels, a protein marker specific to the ER. **(m)** iPSC-differentiated cells were transduced by *AAV2-BR1-GFP* virus (scale bar, 50 μm). **(n)** Western blot analysis to test the specificity of anti-LRP1 antibody using *LRP1* siRNA-transfected iECs. **(o)** Immunostaining analysis to test the specificity of anti-LRP1 antibody using *LRP1* siRNA-transfected iECs with no detergent treatment (scale bar, 20 μm). Results (b, c, e, g, h, l, m, n, o) were reproduced in three independent experiments. Data (a, k) were obtained from three independent experiments. Data in this figure are shown as mean ± SD. Two tailed unpaired t-test: ns, no significance.

Supplementary Fig. 7

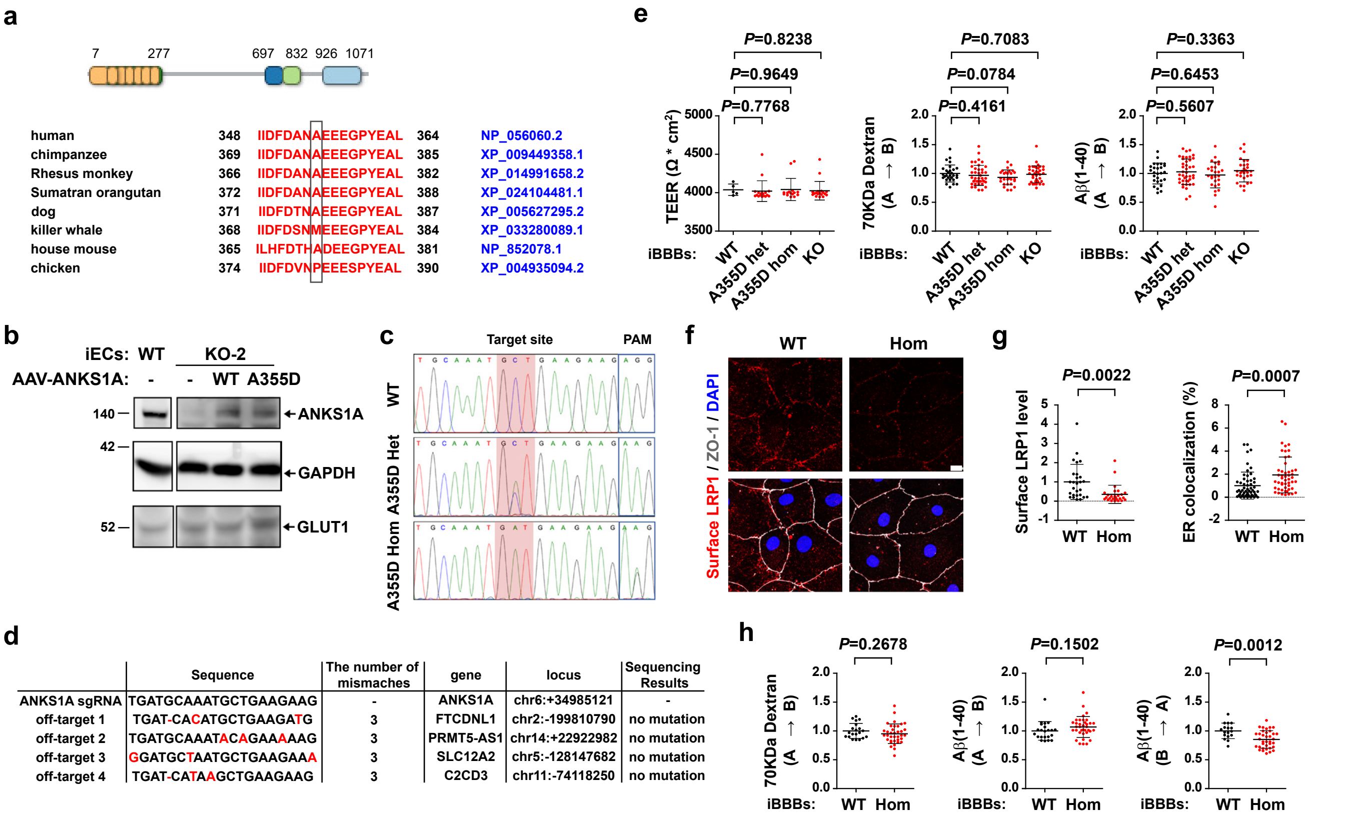

Supplementary Figure 7. Functional analysis of ANKS1A A355D in iPSCs-derived endothelial cells and BBBs (related to Fig. 7).
(a) A multiple ANKS1A protein sequence alignment using COBALT (Constraint Based Alignment Tool, NCBI). **(b)** Western blot analysis showing ANKS1A expression in the AAV-transduced iECs. The human iPSC-derived cells were obtained from the GM23720 cell line. Results were reproduced in three independent experiments. **(c)** Validation of the mutations introduced to the 9th exon of ANKS1A using Sanger sequencing. **(d)** Validation with the potential off-target sites for the sgRNA used in the CRISPR knock-in experiment. **(e)** Paracellular permeability experiments using iBBBs derived from the iECs. The human iPSC-derived cells were generated from the AG09173 cell line. *n*=5, 18, 15, 18 for each group (first panel); *n*=29, 41, 27, 37 for each group (second panel); *n*=27, 41, 27, 29 for each group (third panel). **(f, g, h)** The experiments were performed as described in Fig. 7g, h, k, except that the iECs used were derived from the iPSC line, GM23720 (scale bars, 10 μm). **(g)** *n*=20, 36 cells for each group (left panel); *n*=55, 50 for each group (right panel). Data were from three independent experiments. **(h)** *n*=27, 26 for each group (first panel); *n*=22, 36 for each group (second panel); *n*=17, 36 for each group (third panel). Data in this figure are shown as mean ± SD. Two tailed unpaired t-test.

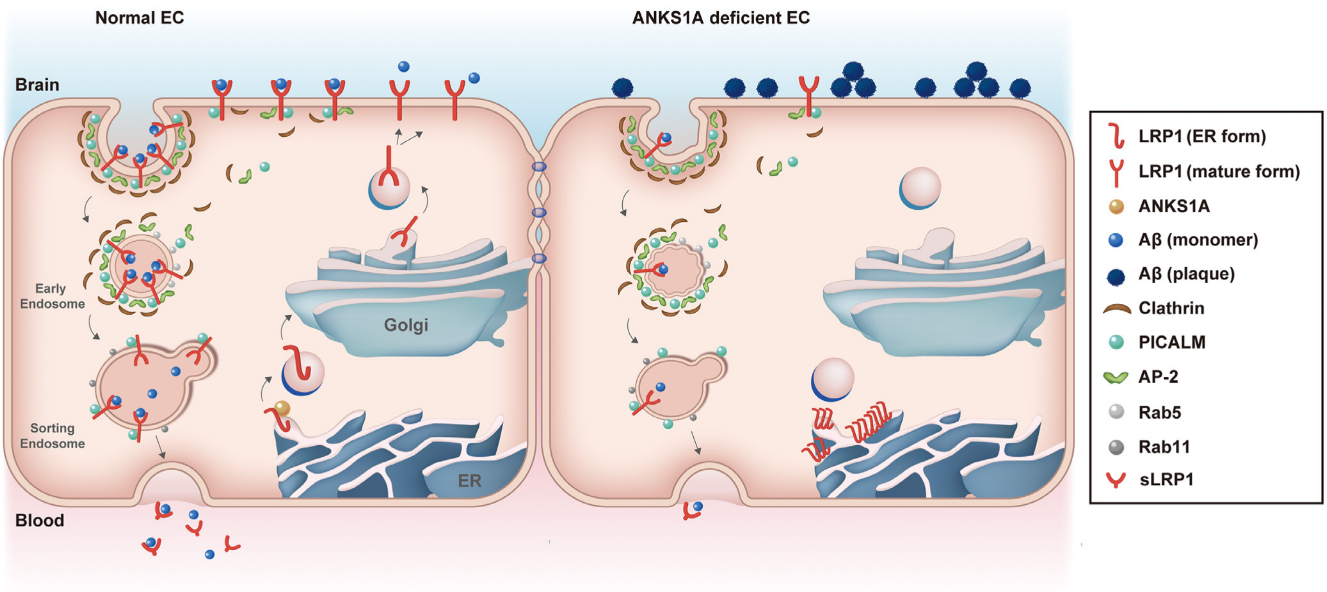

Supplementary Figure 8. Model of ANKS1A deficiency increasing the CAA-associated AD pathogenesis via ineffective LRP1-mediated Aβ clearance.
In normal brain endothelial cells, ANKS1A interacts with the NPXY motifs of LRP1 and facilitates LRP1 export from the ER. LRP1 is cleaved into two fragments in the Golgi apparatus and is then transported into the plasma membrane as a noncovalently associated heterodimeric mature complex. Mature LRP1 binds to Aβ peptides in the cerebrovascular space; this leads to one YXXL motif in its cytoplasmic region interacting with PICALM for inducing clathrin-mediated endocytosis. The endocytosed LRP1 and Aβ peptides are further guided to the apical surface via the RAB5/RAB11-mediated transcytotic pathway. Eventually, LRP1 is shed as a soluble form (sLRP1) and released into the bloodstream together with the bound Aβ peptides. In absence of a functional ANKS1A, the surface levels of mature LRP1 are markedly reduced, thereby decreasing the Aβ clearance. As a result, Aβ peptides accumulate in the microvessels and form neurotoxic plaques to disrupt the cerebrovascular and BBB integrity. We hypothesize that ANKS1A deficiency is a risk factor for CAA-associated AD pathology. The illustration was created with Biorender.com

Supplementary Table 1: List of differentially expressed genes in human iPSC-derived cells

	iASs_1	iASs_2	iASs_3	iASs_4	Average	iEC_1	iEC_2	iEC_3	iEC_4	Average
TJP1	16.9667	16.3789	18.2257	19.0542	17.65638	16.8611	18.2973	22.7919	28.4012	21.58788
ELOVL7	0.034532	0.032694	0.026604	0.180022	0.068463	1.00187	0.679014	0.972918	0.559015	0.803204
CLDN5	1.19419	1.52476	5.20603	0.061831	1.996703	0.018161	0.07341	0.136528	0.049735	0.069458
CTSH	3.95758	6.54198	2.50332	3.4534	4.11407	41.851	26.6112	25.1402	9.64226	25.81117
SLC7A5	39.4095	33.5924	41.4743	82.0406	49.1292	51.2193	62.7448	64.038	170.147	87.03728
PTGIS	0.116154	23.5637	6.70424	1.94488	8.082244	38.0328	32.0975	52.2918	63.4368	46.46473
KDR	0.414493	0.44657	0.302742	10.3519	2.878926	201.414	158.291	133.004	14.6793	126.8471
VWF	0.019394	0.00459	0.041072	0.090936	0.038998	0.008903	0.012814	0.011926	0.049032	0.020669
RTP3	0.067208	0	0	0	0.016802	0	0	0	0	0
OCLN	0.483572	1.26669	0.484195	1.42061	0.913767	2.90908	2.58458	3.48873	2.06599	2.762095
APLNR	0.011375	0.01077	0.043822	0	0.016492	0.804652	0.952072	1.07321	0.090053	0.729997
LRIG1	8.50898	19.8281	21.3246	32.6546	20.57907	6.23211	4.43481	4.99868	4.4685	5.033525
PYGB	79.5239	46.868	62.1613	154.648	85.8003	12.3331	14.1434	17.6951	34.3976	19.6423
HTRA1	137.409	116.696	109.424	212.908	144.1093	48.6233	28.9497	8.32199	17.2889	25.79597
IGFBP2	344.591	615.856	458.28	554.568	493.3238	130.106	108.134	93.1145	183.734	128.7721
MGST1	114.191	50.1002	84.9556	63.9251	78.29298	26.7111	35.8965	84.3322	44.2578	47.7994
CD44	324.154	48.0672	164.083	134.664	167.7421	5.01183	5.78578	4.22338	18.4071	8.357023

	iPCs_1	iPCs_2	iPCs_3	iPCs_4	Average	iEC_1	iEC_2	iEC_3	iEC_4	Average
CDH5	0.020854	0.0212	0	0	0.010513	0.01956	0.037519	0.218194	0.752431	0.256926
THBD	10.9657	55.7838	7.2442	2.95633	19.23751	84.4199	67.0224	73.8312	14.5149	59.9471
PECAM1	0.028892	0	0	0	0.007223	0.009033	0	0.032248	0.012414	0.013424
LSR	6.33656	17.2213	9.25634	3.50265	9.079213	31.0137	32.4092	52.2992	27.9037	35.90645
TMEM154	0.678788	1.09051	0.521897	0.438781	0.682494	0.573059	0.62274	1.02244	0.891213	0.777363
S1PR3	80.0121	27.3474	24.5338	78.3462	52.55988	5.80637	4.53926	4.47812	4.86321	4.92174
CSPG4	18.8627	14.1407	9.52204	14.9958	14.38031	9.73831	7.80297	10.0828	2.48497	7.527263
LINGO1	13.3913	32.605	20.7193	18.2378	21.23835	2.79049	3.52342	4.39376	3.23132	3.484748
GGT1	9.5483	5.48662	9.09372	10.4602	8.64721	3.3564	2.88194	5.26633	0.904936	3.102402
KCNJ8	2.15001	14.0336	2.67557	2.70395	5.390783	0.403408	0.453903	0.453494	0.576529	0.471834
CNN1	25.3084	32.6968	48.1284	73.4833	44.90423	20.7327	24.8999	21.1503	305.029	92.95298
DES	5.89423	2.01208	6.85005	5.54802	5.076095	9.89753	7.61729	3.77655	19.8362	10.28189
FOXD1	1.91939	1.56995	1.91931	1.46119	1.71746	0.222771	0.071168	0.479932	0.355814	0.282421
SEBOX	0	0	0	0	0	0	0.097374	0.090275	0	0.046912
HRC	0.936921	0.076237	2.4657	2.41763	1.474122	0.140636	0.084248	0.14106	0.096306	0.115563
DLK1	210.831	96.0628	759.506	101.237	291.9092	329.497	141.393	21.2604	46.3863	134.6342

Supplementary Table 2 : A description of the clinical characteristics of the participants

The clinical characteristics of the subjects	
sex	M : F = 12 : 27
onset age, y-old	58.3 ± 5.0
duration of AD, y	1.9 ± 1.1
MMSE	18.3 ± 6.9
CDR	1.2 ± 0.8
CDR-SOB	5.5 ± 5.5
Aβ42, pg/mL	321.7 ± 93.1
tTau, pg/mL	788.1 ± 940.4
pTau181, pg/mL	77.1 ± 28.6
pTau/Aβ42 ratio	0.3 ± 0.2
tTau/Aβ42 ratio	2.8 ± 4.3
ApoE4 carrier	18

CDR, clinical dementia rating scale; CDR-SOB, CDR-sum of boxes score; MMSE, mini-mental state examination
