# Supplementary material for: ANKS1A regulates LDL receptor-related protein 1 (LRP1)-mediated cerebrovascular clearance in brain endothelial cells
Source: Nat Commun. 2023 Dec 20;14:8463. doi: 10.1038/s41467-023-44319-3 (PMC10733300; doi:10.1038/s41467-023-44319-3)
Supplement: Supplementary file 3 — Description of Additional Supplementary Files [file 41467_2023_44319_MOESM3_ESM.docx]

**Description of Additional Supplementary Files**

**Supplementary Movie 1:** Cerebral amyloid angiopathy (CAA) in Collagen IV-stained vessels

**Supplementary Data 1:** The analysis of genetic variants of ANKS1A in the studied population

**Supplementary Data 2:** Sequences of siRNAs and oligonucleotides used for this study

**Supplementary Data 3:** The antibodies, proteins and softwares used for this study
