# Supplementary material for: ANKS1A regulates LDL receptor-related protein 1 (LRP1)-mediated cerebrovascular clearance in brain endothelial cells
Source: Nat Commun. 2023 Dec 20;14:8463. doi: 10.1038/s41467-023-44319-3 (PMC10733300; doi:10.1038/s41467-023-44319-3)
Supplement: Supplementary file 8 — Reporting Summary [file 41467_2023_44319_MOESM8_ESM.pdf]

nature portfolio

Corresponding author(s): Soochul Park

Last updated by author(s): Nov 25, 2023

Reporting Summary

Nature Portfolio wishes to improve the reproducibility of the work that we publish. This form provides structure for consistency and transparency in reporting. For further information on Nature Portfolio policies, see our Editorial Policies and the Editorial Policy Checklist.

Statistics

For all statistical analyses, confirm that the following items are present in the figure legend, table legend, main text, or Methods section.

n/a	Confirmed	
☐	☒	The exact sample size (*n*) for each experimental group/condition, given as a discrete number and unit of measurement
☐	☒	A statement on whether measurements were taken from distinct samples or whether the same sample was measured repeatedly
☐	☒	The statistical test(s) used AND whether they are one- or two-sided<br>*Only common tests should be described solely by name; describe more complex techniques in the Methods section.*
☒	☐	A description of all covariates tested
☐	☒	A description of any assumptions or corrections, such as tests of normality and adjustment for multiple comparisons
☐	☒	A full description of the statistical parameters including central tendency (e.g. means) or other basic estimates (e.g. regression coefficient) AND variation (e.g. standard deviation) or associated estimates of uncertainty (e.g. confidence intervals)
☐	☒	For null hypothesis testing, the test statistic (e.g. *F*, *t*, *r*) with confidence intervals, effect sizes, degrees of freedom and *P* value noted<br>*Give P values as exact values whenever suitable.*
☒	☐	For Bayesian analysis, information on the choice of priors and Markov chain Monte Carlo settings
☒	☐	For hierarchical and complex designs, identification of the appropriate level for tests and full reporting of outcomes
☐	☒	Estimates of effect sizes (e.g. Cohen's *d*, Pearson's *r*), indicating how they were calculated

Our web collection on statistics for biologists contains articles on many of the points above.

Software and code

Policy information about availability of computer code

Data collection	LSM700 (Carl Zeiss Microscopy), Axio Zoom.V16 (Carl Zeiss Microscopy), SpectraMaz i3x (Molecular DEVICES), LAS-4000 (Fuji Film)
Data analysis	ZEN black (3.0) software, ZEN blue (3.3) software, ImageJ (Fiji) software, IMARIS (9.7.1) software, Angiotool (0.6a) software, Smart (v3.0.06) software, GraphPad Prism (9.0.1), Premiere Pro Adobe (2019)

For manuscripts utilizing custom algorithms or software that are central to the research but not yet described in published literature, software must be made available to editors and reviewers. We strongly encourage code deposition in a community repository (e.g. GitHub). See the Nature Portfolio guidelines for submitting code & software for further information.

Data

Policy information about availability of data

All manuscripts must include a data availability statement. This statement should provide the following information, where applicable:

- Accession codes, unique identifiers, or web links for publicly available datasets
- A description of any restrictions on data availability
- For clinical datasets or third party data, please ensure that the statement adheres to our policy

Source data are available for graphs plotted in Fig. 1-7 and Supplementary Figs. 1–7. Scans of the full western blot gels can be found in Source data. All raw and analyzed sequencing data can be found at the NCBI Sequence Read Archive (accession number: GSE220105). All other data are available from the corresponding author upon reasonable request. The data that support the finding of this study are openly available in figshare with DOI : 10.6084/m9.figshare.24590481.

Human research participants

Policy information about studies involving human research participants and Sex and Gender in Research.

Reporting on sex and gender	This study is biological one, so we used the word of "sex". The sex, number (m : f number) and age (mean standard deviation) of the participants were described in Supplementary Table 2; M : F = 12 : 27; 58.3 ± 5.0 year-old. The distribution of the rs6930932 variant was comparable between male and female (Supplementary Table 3). The sex of the individuals in our country was determined by ID systems based on birth certificate by the doctors.
Population characteristics	Korean populations with early onset Alzheimer's disease (EOAD) that develops before the age of 65 years. The clinical characteristics of the participants are described in Supplementary Table 2.
Recruitment	The authors used the gene data base for this study that were obtained form the subjects who were recruited consecutively from four university hospitals under the approval of their respective institutional review boards (IRBs). All the subjects had passed both the clinical and cerebrospinal fluid biomarker criteria for AD. All participants and their caregivers (in cases of dementia) gave a written informed consent.
Ethics oversight	Ajou University (AJIRB-BMR-SMP-18-545) and Soonchunhyang University (SCHBC_IRB_2012-124).

Note that full information on the approval of the study protocol must also be provided in the manuscript.

Field-specific reporting

Please select the one below that is the best fit for your research. If you are not sure, read the appropriate sections before making your selection.

- [x] Life sciences
- [ ] Behavioural & social sciences
- [ ] Ecological, evolutionary & environmental sciences

For a reference copy of the document with all sections, see nature.com/documents/nr-reporting-summary-flat.pdf

Life sciences study design

All studies must disclose on these points even when the disclosure is negative.

Sample size	Sample size was determined to be based on the magnitude and consistency of measurable differences between groups. For statistical significance, at least three independent experiments were subjected.
Data exclusions	No data were excluded.
Replication	Each experiment presented in the paper was repeated in multiple times and/or across multiple animals. Replicate experiments were successful. The precise number of repeats are given in the figure legend.
Randomization	All animals were randomized for their genotype information. Throughout this study, both genders were used. However, the AD pathology in 5XFAD mice is higher in females than in males. Therefore, we did not mix genders in the experiments involving 5XFAD mice. The AD pathology-related experiments were conducted mainly on male mice to control for this gender difference, particularly in Fig. 3 and Fig. 5.
Blinding	All investigators were blinded to group allocation during data collection and analysis.

Reporting for specific materials, systems and methods

We require information from authors about some types of materials, experimental systems and methods used in many studies. Here, indicate whether each material, system or method listed is relevant to your study. If you are not sure if a list item applies to your research, read the appropriate section before selecting a response.

Materials & experimental systems

n/a	Involved in the study	
[ ]	[x]	Antibodies
[ ]	[x]	Eukaryotic cell lines
[x]	[ ]	Palaeontology and archaeology
[ ]	[x]	Animals and other organisms
[ ]	[x]	Clinical data
[x]	[ ]	Dual use research of concern

Methods

n/a	Involved in the study	
[x]	[ ]	ChIP-seq
[x]	[ ]	Flow cytometry
[x]	[ ]	MRI-based neuroimaging

Antibodies

Antibodies used

The following primary antibodies were used : rat IgG anti PDGFR-B (1:500, eBioscience, 50-112-2656), rabbit IgG anti-Laminin (1:500, Sigma, L9393), goat IgG anti-Collagen IV (1:500, Southern Biotech, 1340-01), mouse IgG1 anti-b-amyloid (1:500, Biolegend, 803001), rabbit anti b-amyloid (1:500, IBL, 18584), rabbit IgG anti-Apoe3 (1:500, Peprotech, 500-P238), rabbit IgG anti-Calnexin (1:500, Proteintech, 66903-1), rabbit IgG anti-LRP1 (1:500, Abcam, ab92544), sheep IgG anti-LRP1 (1:100, R&D systems, AF4824), rat IgG1 anti-HA (1:100, Sigma, H9658), mouse IgG2b anti-Calregulin (1:250, Santacruz, sc-373863), rabbit IgG anti-Actin (1:3000, Sigma, A2066), mouse IgG1 anti-ZO-1-488 (1:500, Invitrogen, MA3-39100), chicken IgY anti-GFP (1:500, Abcam, ab13970), rabbit IgG anti-GST (1:1000, Homemade), mouse IgG2a anti-His (1:250, Sigma, H1029), rabbit anti-Glut1 (1:500, Merck, 07-1401 / 1:1000, Abcam, ab15309), goat IgG anti-CD144 (1:500, R&D systems, AF938), mouse IgG2a anti-SMA (1:500, R&D systems, MAB1420), rabbit IgG anti-SM22A (1:500, Abcam, ab14106), rabbit IgG anti-AQP4 (1:500, Merck, ab3594), rabbit IgG anti-ANKS1A (1:100, Bethyl, A303-049A / A303-050A), rabbit IgG anti-RAB11A (1:1000, CST, 2413), rabbit IgG anti-RAB11B (1:1000, CST, 2414), rabbit IgG anti-RAB11 (1:100, Santa Cruz, sc-6565), rabbit IgG anti-Occludin (1:3000, Invitrogen, 71-1500), chicken IgY anti-b-Gal (1:250, Abcam, ab9361), rabbit IgG anti-GFAP (1:250, Dako, Z0334), rabbit IgG anti-IBA-1 (1:250, Wako, 019-19741), rabbit IgG anti-Olig2 (1:250, Merck, AB9610), mouse IgG1 anti-GM130 (1:200, BD biosciences, 610822), mouse IgG1 anti-SMI-312 (1:250, Biolegend, 837904), rabbit IgG anti-NeuN (1:250, Merck, ABN78), mouse IgG1 anti-S100b (1:200, Sigma, S2532), mouse IgG1 anti-RAP (1:1000, Santa Cruz, sc-515625), mouse IgG2b anti-Ribophorin I (1:2000, Santa Cruz, sc-48367), goat IgG anti-Ribophorin I (1:2000, Santa Cruz, sc-12164). The list of antibodies are also described in Supplementary Table 5.

Validation

Anks1a antibody (Bethyl, Cat. #A303-050A, Cat. #A303-049A) was validated in Anks1a KO cell lines as shown in Supplementary Fig. 4b, https://www.thermofisher.com/antibody/product/ANKS1A-Antibody-Polyclonal/A303-050A, https://www.thermofisher.com/antibody/product/ANKS1A-Antibody-Polyclonal/A303-049A

LRP1 antibody (Abcam, Cat. #ab92544) were validated in LRP1 conditional KO mouse (PubMed: 33533918), https://www.abcam.com/products/primary-antibodies/lrp1-antibody-epr3724-ab92544.html-amyloid

antibody (Biolegend, Cat. #803001) was validated in controls and patient with Alzheimer's disease (PubMed: 7699397), Purified anti-beta-Amyloid, 1-16 Antibody anti-beta-Amyloid - 6E10 (biolegend.com)-amyloid

antibody (IBL, Cat. #18584) was validated with docosahexaenoic acid treated A(1-42) (PubMed: 19686246), https://www.ibl-america.com/amyloid-beta-n-a-anti-human-rabbit-igg-affinity-purify-1/

Rab11a antibody (CST, Cat. #2413) was validated in Rab11a-specific siRNA-induced knockdown RAW-D cells (PubMed: 33142674), https://www.cellsignal.com/products/primary-antibodies/rab11a-antibody/2413

Rab11b antibody (CST, Cat. #2414) was validated in Rab11b-specific siRNA-induced knockdown RAW-D cells (PubMed: 33302495), https://www.cellsignal.com/products/primary-antibodies/rab11b-antibody/2414-gal

antibody (Abcam, Cat. #ab9361) was validated in Pax2-Cre;R26floxstop-lacZ mice (PubMed: 24223744), https://www.abcam.com/products/primary-antibodies/beta-galactosidase-antibody-ab9361.html

GFP antibody (Abcam, Cat. #ab13970) was validated in transgenic recombinants of an attenuated pseudorabies virus, PRV-152 injected mice (PubMed: 16571749), https://www.abcam.com/products/primary-antibodies/gfp-antibody-ab13970.html

HA antibody (Sigma, Cat. #11867423001) was validated in pcDNA-pro-IL-1-HA transfected HT1080 cells (PubMed: 19651869), https://www.sigmaaldrich.com/KR/ko/product/roche/roahaha

GST antibody (Homemade) was validated with GST or GST-EphA8-JM fusion proteins (PubMed: 17875921).

RAP antibody (Santa Cruz, Cat. #sc515625) was validated in human RAP transfected Cos-1 cells (PubMed: 10504332), https://www.scbt.com/p/rap-antibody-e-7?gad_source=1&gclid=CjwKCAiAsIGrBhAAEiwAEzMlC327bFheFgao7jMrJEef4z1n7dAOv7VlV5Uhu75QvNobao931GoMfhoCELcQAvD_BwE

APOE3 antibody (Peprotech, Cat. #500-P238), https://www.peprotech.com/en/anti-human-apoe3

PDGFR-antibody (eBioscience, cat. #50-112-2656), https://www.fishersci.com/shop/products/cd140b-pdgfrb-monoclonal-antibody-apb5-ebioscience-invitrogen/501122656

PDGFR-antibody (Cell signaling, Cat. #3169), https://www.cellsignal.com/products/primary-antibodies/pdgf-receptor-b-28e1-rabbit-mab/3169

Laminin antibody (Sigma, Cat. #L9393), https://www.sigmaaldrich.com/KR/ko/substance/antilamininantibody1234598765?gclid=CjwKCAiAsIGrBhAAEiwAEzMlC0EqnyD_g4P9_rLVkumCxIG0FcA_UzogoRsjmiKlwDoNOEMLcMUeEhoCAaoQAvD_BwE

Collagen IV antibody (Southern Biotech, Cat. #1340-01), https://www.southernbiotech.com/goat-anti-type-iv-collagen-unlb-1340-01

CD31 antibody (BD biosciences, Cat. #550274), https://www.bdbiosciences.com/en-us/products/reagents/flow-cytometry-reagents/research-reagents/single-color-antibodies-ruo/purified-rat-anti-mouse-cd31.550274

Calnexin antibody (Proteintech,, Cat. #66903-1), https://www.ptglab.com/products/Calnexin-Antibody-66903-1-Ig.htm

HA antibody (Sigma, Cat. #H9658), https://www.sigmaaldrich.com/KR/ko/product/sigma/h9658

Calregulin antibody (Santa Cruz, Cat. #sc373863), https://www.scbt.com/p/calregulin-antibody-f-4

Actin antibody (Sigma, Cat. #A2066), https://www.sigmaaldrich.com/KR/ko/product/sigma/a2066?gclid=CjwKCAiAsIGrBhAAEiwAEzMlCx_1GGurOx1H9aa3uJfaN5defGUw8rm9AvhFAIfIFdxBPDrobUei3hoCMB0QAvD_BwE

ZO-1 antibody (Invitrogen, Cat. #MA3-39100), https://www.thermofisher.com/antibody/product/ZO-1-Antibody-clone-ZO1-1A12-Monoclonal/MA3-39100-A488

Glut1 antibody (Merck, Cat. #07-1401), https://www.merckmillipore.com/KR/ko/product/Anti-GLUT-1-Antibody-CT,MM_NF-07-1401?ReferrerURL=https%3A%2F%2Fwww.google.com%2F

(Abcam, Cat. #ab15309), https://www.abcam.com/en-se/products/primary-antibodies/glucose-transporter-glut1-antibody-ab15309

CD144 antibody (R&D systems, Cat. #AF938), https://www.rndsystems.com/products/human-ve-cadherin-antibody_af938?gad_source=1&gclid=CjwKCAiAsIGrBhAAEiwAEzMlC5FadJy784NlFM3mH8KK7INpx0_XkG7RswF_rVO3ESLwiZQCD5BzQhoCfHsQAvD_BwE&gclsrc=aw.ds

SMA antibody (Sigma, Cat. #C6198), https://www.sigmaaldrich.com/KR/ko/substance/antiactinasmoothmusclecy3antibodymousemonoclonal1234598765?gclid=CjwKCAiAsIGrBhAAEiwAEzMlCyK8ywdMs_5f0YRiZmrqEQGUZqkp9YFkZAwGr9pcZUJxcNvuNpBJ8hoCbIEQAvD_BwE

SM22A antibody (Abcam, Cat. #ab14106), https://www.abcam.com/products/primary-antibodies/taglntransgelin-antibody-ab14106.html

AQP4 antibody (Merch, ab3594), https://www.merckmillipore.com/KR/ko/product/Anti-Aquaporin-4-Antibody-CT,MM_NF-AB3594-50UL
Occludin antibody (Invitrogen, Cat. #71-1500), https://www.thermofisher.com/antibody/product/Occludin-Antibody-Polyclonal/71-1500
GFAP antibody (Dako, Cat. #Z0334), https://www.agilent.com/en/product/immunohistochemistry/antibodies-controls/primary-antibodies/glial-fibrillary-acidic-protein-(concentrate)-76683
IBA1 antibody (Wako, Cat. #019-19741), https://labchem-wako.fujifilm.com/us/product/detail/W01W0101-1974.html
Olig2 antibody (Merck, Cat. #AB9610), https://www.sigmaaldrich.com/KR/ko/product/mm/ab9610?gclid=CjwKCAiAsIGrBhAAEiwAEzMlC7XhKWPRiy6xmt8dQ1p0WjXysUAJjbDitThK4ooDqPKFx09HxG6sEBoCkMoQAvD_BwE
GM130 antibody (BD biosciences, Cat. #610822), https://www.bdbiosciences.com/ko-kr/products/reagents/microscopy-imaging-reagents/immunofluorescence-reagents/purified-mouse-anti-gm130.610822
SMI-312 antibody (Biolegend, Cat. #837904), https://www.biolegend.com/en-us/explore-new-products/purified-anti-neurofilament-marker-pan-axonal-cocktail-12811?GroupID=BLG15643
NeuN antibody (Merck, Cat. #ABN78), https://www.sigmaaldrich.com/KR/ko/product/mm/abn78?gclid=CjwKCAiAsIGrBhAAEiwAEzMlC5G6xLX9haaCPRT1Co0kQYE-TyBNPfg-mneTBI6Qnztu_DGt0ArZmRoC1J8QAvD_BwE
S100b antibody (Sigma, Cat. #S2532), https://www.sigmaaldrich.com/KR/ko/product/sigma/s2532
Ribophorin antibody (Santa Cruz, Cat. #sc48367), https://www.scbt.com/ko/p/ribophorin-i-antibody-e-7?requestFrom=search

Eukaryotic cell lines

Policy information about cell lines and Sex and Gender in Research

Cell line source(s)	The human iPSC lines (GM23720 and AG09173) were from the Coriell Institute for Medical Research. The HEK293 cells CRL-1573) and bEnd.3 cells (CRL-2299) were obtained from ATCC.
Authentication	bEnd.3 cells were confirmed to be brain endothelial cells based on the expression of specific endothelial markers shown in Fig. 1d and Supplementary Fig. 1k-o. The RT-qPCR primers for bEnd.3 cells in Supplementary Fig. 1h and j are shown in Supplementary Table 4. HEK293 cells were confirmed using the endogenous expression of ANKS1A, Calnexin, and Calregulin proteins shown in Fig. 4f, h and Supplementary Fig. 4b, d. The RT-qPCR primers for human iPSC lines are shown in Supplementary Table 4.
Mycoplasma contamination	PCR-based mycoplasma testing was routinely performed and all cell lines used for this study were found negative for the contamination.
Commonly misidentified lines (See ICLAC register)	No commonly misidentified cell lines were used in this study.

Animals and other research organisms

Policy information about studies involving animals; ARRIVE guidelines recommended for reporting animal research, and Sex and Gender in Research

Laboratory animals	Mice were housed in plastic cages on a 12 h light/dark cycle with ad libitum access to water and a standard laboratory diet. Room ambient temperature was set between 20-25 degrees Celsius and humidity was kept between 30-70%. Laboratory mice were maintained on a congenic C57BL/6 background. Both male and female mice were used in this study. AAV injection experiments were conducted on 2-month-old mice. The mice with 5XFAD background were sacrificed at 2-7 months of age for this study. For aging study, only wild type mice were used and sacrificed at 18-22 months of age. Anks1a+/lacZ gene trap mice have been previously described, (Kim, J. et al., 2010). Anks1af/f mice have been previously described, (Ryu, H. et al., 2021). 5XFAD mice have been previously described, (Oakley, H. et al., 2006). Tie2-Cre mice have been previously described, (Kisanuki, Y. Y. et al., 2001).
Wild animals	No wild type animals were used in this study.
Reporting on sex	For the experiments involving 5XFAD mice, we have used male mice since female 5XFAD mice often reveal more severe AD pathology. We did not mix genders for the experiments; N=5 for both Tie2-Cre;5XFAD and ANKS1Af/f;Tie2-Cre;5XFAD mice or N=3 for ANKS1A+/f;Tie2-Cre;5XFAD mice in Fig. 3 and Supplementary Fig. 3; N=5 for both 5XFAD and ANKS1A-/-; 5XFAD mice or N=3 for ANKS1A+/-; 5XFAD mice in Fig. 5 and Supplementary Fig. 5. In a separate experiment, we also performed AD pathological analysis for female mice; N=3 mice for each group in Supplementary Fig. 5c-f.
Field-collected samples	No field-collected samples were used in this study.
Ethics oversight	All experiments were approved by and were in compliance with the Sookmyung Women's University Institutional Animal Care and Use Committee (SWU-IACUC-2104-002-1). All mice were housed and handled at the animal facility of Sookmyung Women's University.

Note that full information on the approval of the study protocol must also be provided in the manuscript.

Clinical data

Policy information about clinical studies

All manuscripts should comply with the ICMJEguidelines for publication of clinical research and a completedCONSORT checklist must be included with all submissions.

Clinical trial registration	KCT0000847, CRIS (clinical research information service (CRIS), Korea Disease Control and Prevention Agency (KDCA))
Study protocol	The study protocol cannot be provided since the data that used for this study is only a tiny part of the study focusing on ANKS1 gene variants.
Data collection	Hospital-based collection, prospectively from January 2014 to January 2017
Outcomes	The study is just a gene analysis focusing on ANKS1A. So the outcome issue is not applicable.
